# Objective verification of continuous speech sound discrimination using the acoustic change complex

**DOI:** 10.3389/fnhum.2026.1771785

**Published:** 2026-06-09

**Authors:** Eun Kyung Jeon, Sarah A. Klemuk, Carolyn J. Brown

**Affiliations:** 1 Department of Communication Sciences and Disorders, University of Iowa, Iowa City, IA, United States; 2 Department of Otolaryngology, University of Iowa, Iowa City, IA, United States

**Keywords:** acoustic change complex, cochlear implant, cortical auditory evoked potential, speech sound discrimination, auditory cortex, Ling six sounds

## Abstract

**Objective:**

To evaluate the feasibility of using the acoustic change complex (ACC) as an objective cortical marker of continuous speech sound discrimination using connected Ling-six stimuli.

**Design:**

Cortical auditory evoked potentials were recorded from 10 young adults with normal hearing using a continuous, pause-free sequence of the six Ling phonemes (/u-a-i-ʃ-s-m/). ACC responses were recorded in a passive listening paradigm with minimal electrodes. Reproducibility was assessed in a subset of participants retested 1 year later.

**Results:**

Robust ACC responses were elicited for all six phonemic transitions in every participant, indicating reliable cortical discrimination within the continuous speech stream. Although response morphology varied across listeners, within-subject responses demonstrated strong reproducibility over a one-year interval (mean cross-correlation = 0.95).

**Conclusion:**

Continuous Ling-six stimuli reliably evoke ACC responses reflecting cortical speech sound discrimination. This efficient and repeatable paradigm shows promise as an objective verification tool for auditory learning and sound discrimination, particularly in populations for whom behavioral assessment is unreliable.

## 1 Introduction

Early cochlear implantation has dramatically improved auditory access and spoken language outcomes for children with severe-to-profound hearing loss. As a result, extraordinary efforts have been made to identify candidates early and to provide cochlear implants (CIs) within critical periods for auditory and language development. Despite these advances, variability in speech and language outcomes remains substantial, even among children implanted at similar ages with comparable devices and access to post-implant intervention (Nicholas and Geers, 2007; Niparko et al., 2010; Sharma et al., 2015). One contributing factor to this variability is the challenge of achieving optimal sound processor programming, particularly in infants, young children, and individuals who are unable to provide reliable behavioral feedback.

During CI programming sessions, audiologists attempt to establish electrical stimulation levels that provide access to soft sounds while maintaining comfort for louder inputs. This process relies heavily on behavioral responses to speech and non-speech stimuli. However, obtaining consistent and interpretable behavioral responses can be difficult or impossible in very young children, children with additional developmental or medical conditions, or individuals with limited attention or communication abilities. In clinical practice, when caregivers report limited auditory responsiveness or lack of emerging speech perception following implantation, clinicians are often faced with difficult decisions, including whether to modify programming parameters, pursue additional diagnostic testing, consider device revision, or continue monitoring while awaiting developmental progress. Objective measures that can inform these decisions and reflect meaningful auditory processing are therefore of high clinical value.

The Daniel Ling Six Sound Test has long been used as a practical tool to assess functional listening skills in children and adults with hearing loss (Ling, 1976; Ling, 1988). The six phonemes (/u/, /a/, /i/, /ʃ/, /s/, and /m/) span a broad range of speech frequencies from approximately 250–8,000 Hz and provide a simple yet powerful representation of access to critical spectral cues. Clinically, the Ling six sounds are widely used by audiologists, parents, and educators to monitor auditory access following hearing aid or CI fitting and to support daily listening checks (Smiley et al., 2004; Schow and Nerbonne, 2013). Detection of these sounds provides information about audibility, while accurate identification and differentiation reflect early stages of speech sound discrimination and auditory learning (Scollie et al., 2012). However, the Ling six sound test is inherently behavioral and therefore limited in its applicability to individuals who cannot reliably respond.

Several objective measures are currently used to support CI programming in difficult-to-test populations. Electrically evoked compound action potentials (ECAPs) and electrically evoked stapedius reflex thresholds (ESRTs) provide valuable information about peripheral auditory nerve and reflex pathway function and are commonly used to estimate appropriate stimulation levels in pediatric CI users. Although relationships between these measures and programming parameters have been reported, correlations with speech perception outcomes are modest and inconsistent (Jeon et al., 2010; van Eijl et al., 2017; Kim et al., 2010; James et al., 2025). This limitation is not surprising, as ECAPs and ESRTs primarily reflect peripheral auditory processing and do not directly assess neural activity at cortical levels where speech perception, discrimination, and auditory learning occur. Consequently, there is a clear need for objective measures that reflect central auditory processing and cortical discrimination of speech-relevant acoustic information.

Cortical auditory evoked potentials (CAEPs) provide a noninvasive means of assessing auditory processing at the level of the auditory cortex. Among CAEP components, the acoustic change complex (ACC) has received increasing attention as an index of cortical sensitivity to changes within ongoing acoustic stimuli. The ACC is characterized by a P1–N1–P2 response complex elicited by a change in spectral, temporal, or intensity features of a continuous sound rather than by sound onset alone (Martin and Boothroyd, 2000; Friesen and Tremblay, 2006). Importantly, the ACC is thought to reflect neural mechanisms underlying sound discrimination rather than simple detection, making it particularly relevant for studying speech perception and auditory learning.

A growing body of research has demonstrated that ACC responses can be elicited by a wide range of acoustic changes, including vowel contrasts, spectral ripple transitions, and changes in speech features, in both normal-hearing and hearing-impaired listeners (Won et al., 2011; Brown et al., 2015; Scheperle and Abbas, 2015). ACCs have also been recorded in children and in CI users using passive listening paradigms, highlighting their potential clinical utility for populations unable to perform behavioral tasks (He et al., 2015; Sharma et al., 2015; Jeon et al., 2023). Moreover, ACC amplitude and morphology have been shown to correspond to behavioral discrimination performance and to change with auditory experience, supporting their interpretation as markers of cortical plasticity and learning (Tremblay et al., 2001; He et al., 2012; Brown et al., 2017; Jeon et al., 2023; Kim, 2015).

Despite this promise, most ACC paradigms used in prior research have relied on discrete stimuli separated by long inter-stimulus intervals, often on the order of several seconds. While such designs are well suited for experimental control, they are time-consuming and poorly aligned with the demands of clinical practice. Additionally, discrete stimulus paradigms do not reflect the continuous nature of everyday speech perception, in which listeners must rapidly detect and interpret acoustic changes within ongoing sound streams. From a learning and neurophysiological perspective, continuous speech engages predictive coding, adaptation, and dynamic cortical updating processes that are central to auditory learning and language development (Näätänen et al., 2007; Bendixen et al., 2012; Sharma and Glick, 2016).

In the present study, we sought to address these limitations by developing a clinically efficient ACC paradigm using the Ling six speech sounds presented in a continuous, connected sequence without inter-stimulus intervals. This approach preserves the spectral relevance and clinical familiarity of the Ling six sounds while more closely approximating natural listening conditions. By minimizing recording time and electrode configuration requirements, this paradigm is designed to be feasible for routine clinical use.

The ultimate goal of this line of research is to establish an objective cortical measure that can support verification of CI sound processor programming and monitoring of auditory learning in young children and individuals with complex needs. As a foundational step, the present report focuses on assessing the feasibility and within-subject reproducibility of ACC responses elicited by continuous Ling six speech sounds in normal-hearing young adults who can behaviorally discriminate the phoneme sequence. Demonstrating reliable cortical discrimination under these conditions is a necessary prerequisite for future application in pediatric and hearing-impaired populations.

## 2 Methods

### 2.1 Subjects

A total of 10 young adults participated in the study: five males and five females (mean age = 25.9 years, SD = 4.87). All participants had normal hearing with air conduction thresholds of 20 dB HL or better from 250 to 8,000 Hz. The Institutional Review Board at The University of Iowa approved this research project, and participants were compensated for their time.

### 2.2 Stimuli

The six Ling phonemes (/u/, /a/, /i/, /ʃ/, /s/, and /m/) were recorded from a single adult female speaker in a sound-treated environment using Adobe Audition software (Adobe Systems, San Jose, CA, United States). A single speaker was selected to minimize variability related to vocal tract characteristics and fundamental frequency across stimuli.

To reduce natural amplitude and frequency fluctuations inherent in connected speech while preserving spectral characteristics relevant for discrimination, a single stable period from each phoneme was extracted and repeated to create a steady-state segment with a duration of 500 ms per phoneme. This approach ensured consistent acoustic structure within each phoneme while maintaining the distinct spectral features necessary for eliciting acoustic change responses at phoneme boundaries.

Linear Predictive Coding (LPC) analysis was performed using Praat software (version 6.0; Amsterdam, The Netherlands) to verify that formant frequencies for each vowel and consonant were consistent with normative values reported for female speakers (Ross et al., 1991; Styler, 2021). All six 500-ms phoneme segments were then concatenated to form a continuous 3-s speech sequence presented in the following fixed order: /u–a–i–ʃ–s–m/. Care was taken to ensure that no audible clicks, transients, or unintended acoustic artifacts occurred at phoneme boundaries.

Prior to electrophysiological recording, participants confirmed that they could clearly perceive and correctly identify all six phonemes within the continuous stimulus sequence.

### 2.3 Stimulus presentation

Stimuli were presented in the sound field at a conversational listening level via a single loudspeaker positioned approximately 1.2 m (four feet) in front of the participant at 0° azimuth. The acoustic signal was routed from a desktop computer through a National Instruments SCB-68 soundcard, a Tucker-Davis Technologies (TDT) attenuator (Austin, TX, United States), and a Grason-Stadler GSI audiometer (Eden Prairie, MN, United States).

Stimulus levels were calibrated in the sound-treated booth using a sound level meter set to A-weighting with slow response, referenced to the center of the participant’s head position. The presentation level was verified at 59 ± 1 dBA SPL, corresponding to a typical conversational speech level. To ensure consistency across stimuli, root-mean-square (RMS) amplitude was verified to be closely matched across all six phonemes, minimizing amplitude-related confounds.

Stimulus presentation and data acquisition were controlled using a custom graphical user interface developed in MATLAB (version 2014b; MathWorks, Natick, MA, United States) with associated functions from the MATLAB Data Acquisition Toolbox. The continuous Ling six stimulus was presented for a total of 200 repetitions (sweeps), delivered in four blocks of 50 sweeps. No inter-stimulus interval was used between stimulus presentations, reflecting a continuous listening paradigm consistent with natural speech perception. Responses were segmented into epochs time-locked to individual phoneme transitions within the continuous stimulus stream, such that each analysis window represents cortical activity surrounding a specific acoustic change rather than a continuous loop.

### 2.4 Electrophysiological recording

Cortical auditory evoked potentials were recorded using surface electrodes placed at the vertex (Cz), left mastoid (M1), and right mastoid (M2), with a forehead electrode serving as ground. Electrode impedances were maintained below 5 kΩ and balanced across recording sites.

Electrophysiological signals were amplified and digitized using the OptiAmp Intelligent Hearing Systems amplifier (Miami, FL, United States) with a gain of 10,000 and bandpass filtering from 1 to 30 Hz. Continuous EEG data were segmented and averaged offline using custom MATLAB scripts.

Artifact rejection criteria were applied to exclude individual sweeps contaminated by excessive noise or movement artifact. To monitor and reject ocular artifacts, additional electrodes were placed above and below one eye to record vertical electrooculogram activity. Sweeps exceeding the predefined artifact threshold were excluded from averaging.

Participants were seated comfortably in a sound-treated booth and instructed to remain awake and relaxed throughout the recording session. To promote a passive listening state while minimizing movement, participants were allowed to watch captioned videos, read magazines, or use silent mobile devices during testing.

Recording of 200 sweeps required approximately 10 min. Including electrode placement, calibration, and short breaks, the total test session duration was less than 40 min per participant, supporting the feasibility of this paradigm for clinical application.

### 2.5 Data analysis

ACC responses were analyzed offline using custom MATLAB scripts. Continuous EEG recordings were segmented relative to each phoneme transition within the connected Ling-six stimulus. For each phonemic boundary, analysis epochs extended from 50 ms prior to the acoustic change to 300 ms following the change to capture the full ACC waveform.

ACC components were identified based on established morphological criteria. The P1 component was defined as the first positive peak occurring approximately 50–80 ms following the acoustic change, followed by the N1 component occurring approximately 90–140 ms post-change, and the P2 component defined as the subsequent positive peak occurring approximately 160–230 ms after the transition. Peak identification was performed visually by experienced investigators to account for variability in waveform morphology associated with continuous speech stimuli.

ACC presence was determined by the identification of a reproducible P1–N1–P2 complex exceeding the noise floor for each phoneme transition. For descriptive purposes, relative response strength across phoneme transitions was assessed qualitatively by comparing N1–P2 peak-to-peak amplitudes within and across participants. Because the primary objective of this study was to assess feasibility rather than to test specific hypotheses, formal inferential statistical comparisons of ACC amplitudes across phoneme transitions were not conducted.

To evaluate within-subject reproducibility, ACC waveforms obtained during Session 1 and Session 2 were compared using point-to-point Pearson correlation between time-aligned waveforms across the full 3-s response window. This approach corresponds to correlation at zero time lag, rather than computation of a full cross-correlation function across multiple lags. Correlation coefficients were calculated for each participant to quantify waveform similarity across sessions.

## 3 Results

### 3.1 ACC responses evoked by continuous Ling-six speech in an individual listener

**Figure 1:**
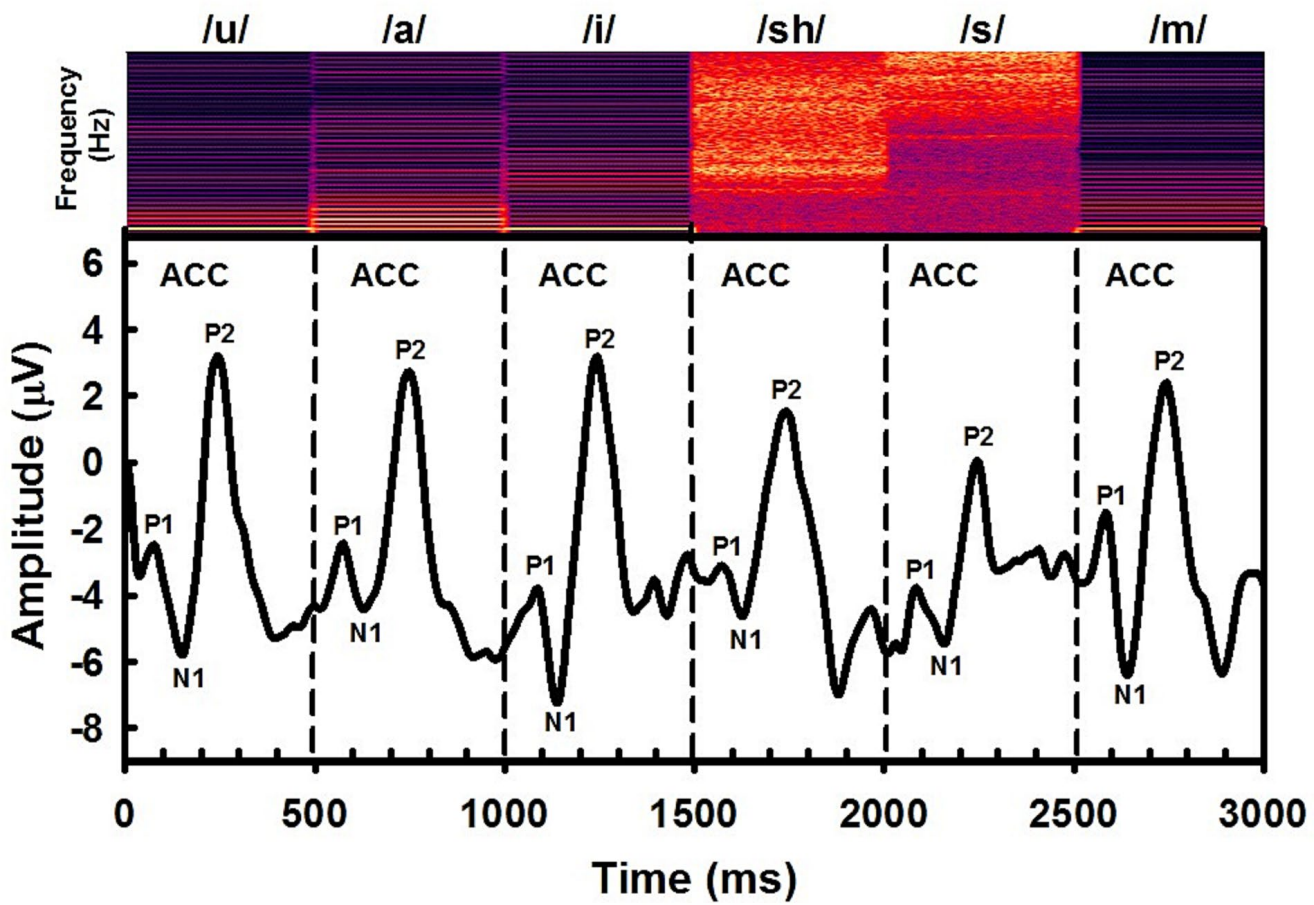
Continuous Ling-six speech stimulus and representative acoustic change complex (ACC) response. The top panel shows the spectrogram of the continuous, pause-free Ling-six speech sequence (/u–a–i–ʃ–s–m/), illustrating distinct spectrotemporal contrasts across phoneme transitions. The bottom panel displays the corresponding ACC waveform from a representative normal-hearing participant, averaged over 200 sweeps. Vertical dashed lines indicate phoneme transition boundaries. Clear P1–N1–P2 complexes are observed following each acoustic change, demonstrating cortical discrimination of speech sounds within a continuous speech stream.

Figure 1 illustrates ACC responses obtained from a representative normal-hearing participant using the continuous Ling-six speech stimulus with no inter-stimulus intervals. The upper panel displays the spectrogram of the connected speech sequence (/u–a–i–ʃ–s–m/), demonstrating clear spectrotemporal contrasts across phoneme transitions. The lower panel shows the corresponding cortical response waveform averaged across 200 sweeps.

Distinct ACC responses were observed following each phonemic transition, characterized by identifiable P1, N1, and P2 components. These responses occurred consistently at each acoustic boundary (e.g., /u/→/a/, /a/→/i/, /i/→/ʃ/), indicating reliable cortical detection of spectrotemporal change within an ongoing speech stream rather than responses to sound onset alone. Across transitions, the P1–N1–P2 complexes were temporally aligned with expected ACC latencies, with P1 occurring approximately 50–80 ms following each transition, N1 occurring between approximately 90–140 ms, and P2 occurring between approximately 160–230 ms post-change.

Notably, ACC morphology varied across phoneme transitions within the same participant. Transitions involving large spectral contrasts, particularly the vowel-to-fricative transition from /i/ to /ʃ/, elicited larger-amplitude P2 responses compared to transitions involving more subtle spectral changes, such as /ʃ/ to /s/. This pattern suggests differential cortical sensitivity to the magnitude and nature of acoustic change within the continuous speech sequence.

### 3.2 Group-level ACC responses and inter-individual variability

**Figure 2:**
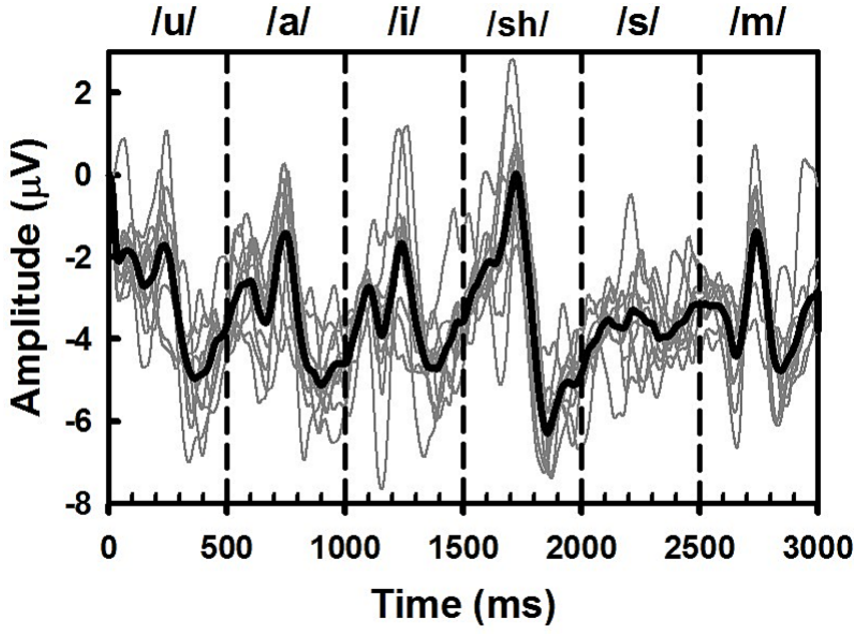
Individual and grand-average ACC responses evoked by continuous Ling-six speech. Gray traces represent individual ACC waveforms from 10 normal-hearing young adult participants, each averaged over 200 sweeps. The bold black trace shows the grand-average waveform across participants. Vertical dashed lines denote phoneme transition boundaries (/u/, /a/, /i/, /ʃ/, /s/, /m/). Although waveform morphology varies across individuals, ACC responses—most prominently the P2 component—are evident at each phonemic transition, indicating reliable cortical discrimination across listeners.

Figure 2 presents ACC responses from all 10 participants, with individual waveforms shown in gray and the grand-average waveform shown as a bold black trace. Each individual waveform represents the average of 200 sweeps for that participant. Vertical dashed lines indicate phoneme transition boundaries within the continuous stimulus.

ACC responses were observed at each phonemic transition for all participants, confirming that the continuous Ling-six stimulus reliably elicited cortical discrimination responses at the group level. However, response morphology exhibited notable inter-individual variability in amplitude and waveform shape, a pattern consistent with previous ACC studies using complex speech stimuli. Despite this variability, ACC components—particularly the P2 peak—were consistently identifiable across participants for all six phoneme transitions.

Inspection of the grand-average waveform revealed systematic differences in ACC magnitude across transitions. The largest P2 amplitudes were observed for the /i/→/ʃ/ transition, corresponding to a substantial shift from a high-front vowel to a broadband fricative. In contrast, the smallest P2 responses were observed for the /ʃ/→/s/ transition, which involves relatively similar fricative spectral characteristics. These findings suggest that ACC amplitude reflects the relative salience of spectrotemporal change within the continuous speech stream.

**Figure 3:**
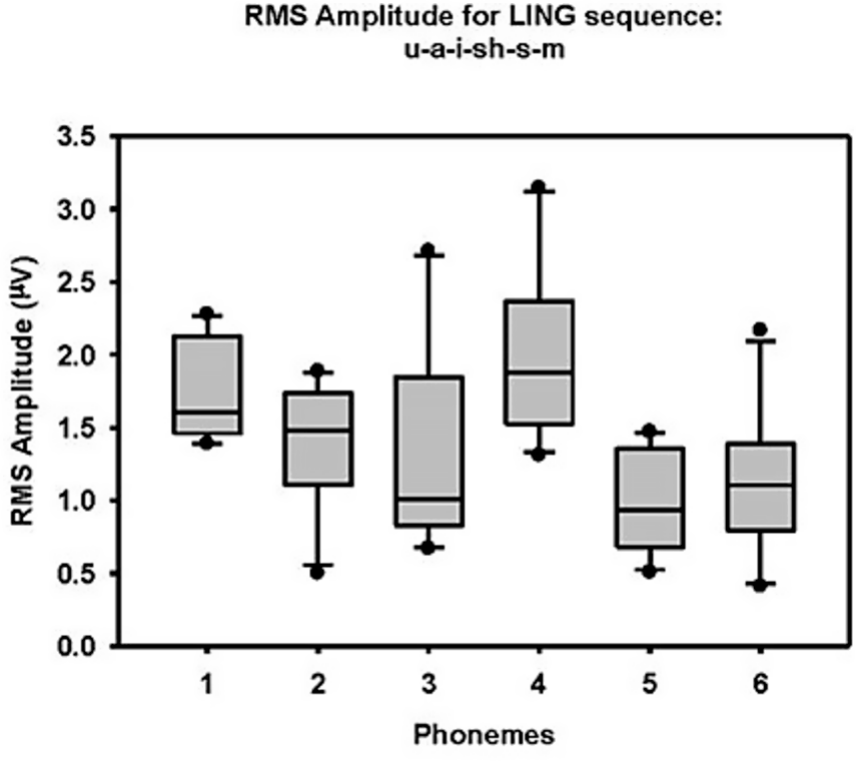
RMS amplitude of ACC responses across phoneme positions in the continuous Ling-six sequence. RMS amplitude was calculated over a 400 ms time window following each phoneme transition. Box plots illustrate the distribution of RMS amplitudes across participants for each phoneme position in the sequence. The central line indicates the median, boxes represent the interquartile range (25th–75th percentile), and whiskers indicate the 10th and 90th percentiles. Dots represent minimum and maximum values.

To further quantify response magnitude, root-mean-square (RMS) amplitude of the ACC response was calculated over a 400 ms time window following each phoneme transition (Figure 3). RMS values differed across phoneme positions in the continuous Ling-six sequence, with some transitions showing higher median response amplitudes and broader variability than others. These results provide quantitative support for differences in ACC response magnitude across transitions within the continuous speech sequence.

To provide a descriptive summary of response strength, peak-to-peak ACC amplitudes (N1–P2) were visually identifiable for all transitions in all participants, with larger amplitudes generally observed for vowel–consonant transitions compared to within-class consonant transitions. While formal inferential comparisons across phoneme transitions were beyond the scope of this feasibility study, these descriptive patterns support the sensitivity of the ACC to graded acoustic contrasts within connected speech.

### 3.3 Within-subject reproducibility of ACC responses over time

**Figure 4:**
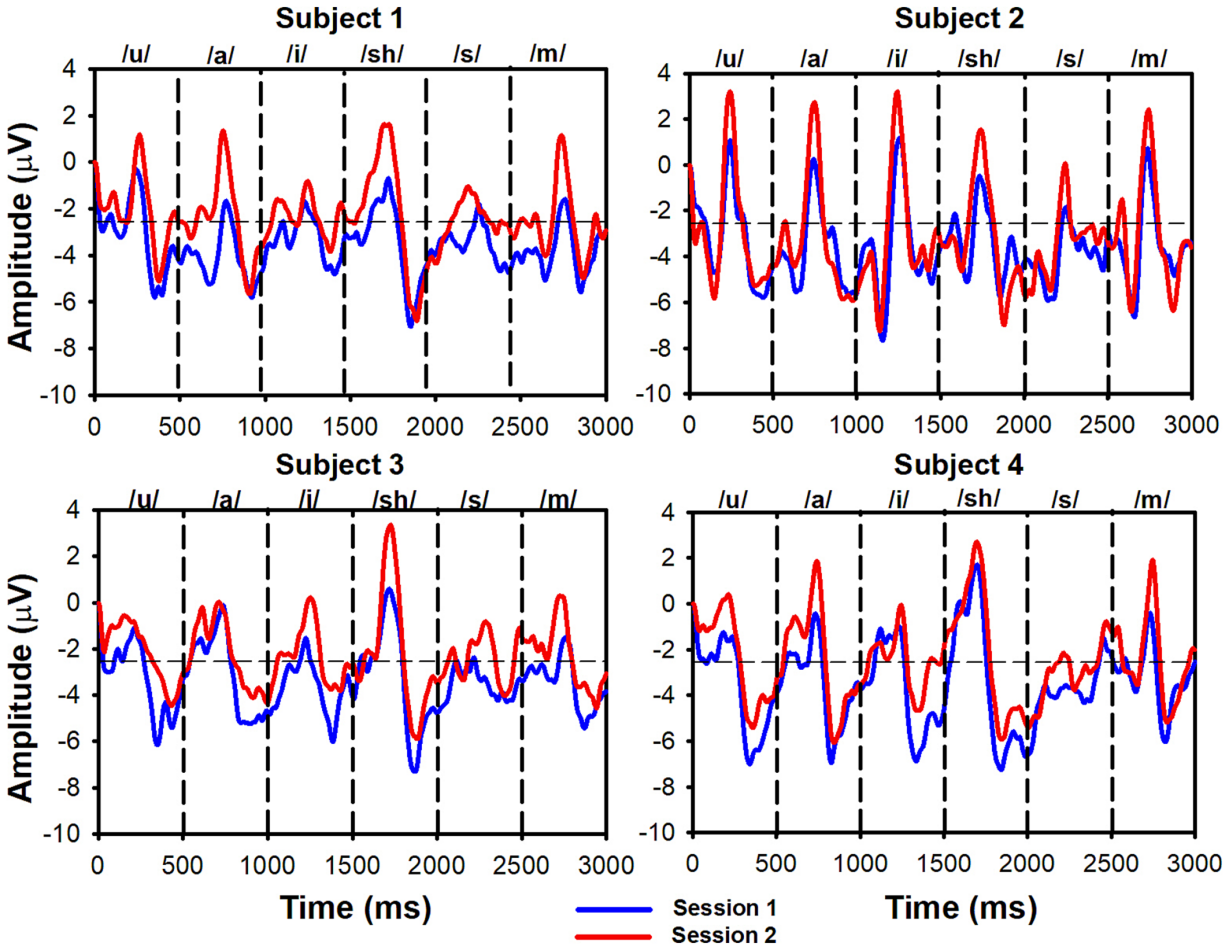
Within-subject reproducibility of ACC responses over a one-year interval. ACC waveforms from four normal-hearing participants recorded during two sessions separated by approximately 1 year. Blue traces correspond to Session 1 and red traces correspond to Session 2. Vertical dashed lines mark phoneme transition boundaries, and the horizontal dashed line represents the noise floor. Within-subject waveform morphology and timing are highly consistent across sessions, demonstrating strong long-term reproducibility of cortical discrimination responses elicited by continuous Ling-six speech.

To assess the stability and reproducibility of ACC responses elicited by the continuous Ling-six stimulus, four participants returned for repeat testing approximately 1 year after their initial session. Figure 4 displays within-subject comparisons for these participants, with Session 1 responses shown in blue and Session 2 responses shown in red. Vertical dashed lines denote phoneme transition boundaries, and the horizontal dashed line represents the noise floor.

Across all four participants, ACC waveforms demonstrated strong qualitative similarity across sessions, with consistent timing and polarity of P1, N1, and P2 components at each phoneme transition. Although minor differences in amplitude were observed between sessions, overall waveform morphology remained highly stable within individuals.

Quantitative assessment of reproducibility was performed using cross-correlation analysis with zero time lag between Session 1 and Session 2 waveforms for each participant. The mean cross-correlation coefficient was 0.948 (SD = 0.01), indicating a high degree of within-subject similarity over a one-year interval. In all cases, ACC peaks and troughs exceeded the noise floor, supporting the robustness of the recorded responses.

These findings demonstrate that ACC responses evoked by continuous Ling-six speech are not only reliably elicited across individuals but also highly stable within individuals over extended periods, underscoring their potential utility for longitudinal monitoring of cortical auditory discrimination and learning.

## 4 Discussion

The present study demonstrates that continuous, connected Ling-six speech stimuli can reliably evoke ACC responses reflecting cortical discrimination of speech sounds in normal-hearing listeners. ACC responses were consistently observed across all phoneme transitions and exhibited strong within-subject reproducibility over a one-year interval. These findings extend prior ACC literature by demonstrating that cortical discrimination can be measured using a continuous, time-efficient, and clinically relevant speech paradigm, addressing key limitations of traditional ACC methodologies.

The presence of time-locked P1–N1–P2 responses at each phoneme transition confirms that the ACC reflects cortical encoding of acoustic change within an ongoing stimulus rather than simple sound detection (Martin and Boothroyd, 2000; Friesen and Tremblay, 2006). The observed variation in ACC morphology across phoneme transitions further supports the interpretation that ACC responses are sensitive to the magnitude and salience of acoustic differences.

Specifically, larger responses observed for vowel-to-fricative transitions likely reflect greater spectrotemporal contrast, whereas smaller responses for within-class consonant transitions suggest reduced neural discrimination demands. This pattern is consistent with prior findings demonstrating that ACC amplitude scales with acoustic change magnitude and perceptual discriminability (Won et al., 2011; Brown et al., 2017). Brown et al. (2017) further showed that individuals with enhanced spectral discrimination abilities exhibit larger ACC responses and that electrophysiologic responses are moderately correlated with behavioral discrimination performance, supporting the interpretation of ACC as a neural marker of auditory discrimination rather than detection.

Importantly, the strong within-subject reproducibility observed over a one-year interval (mean cross-correlation = 0.95) indicates that ACC responses are stable over time. Previous studies have demonstrated favorable test–retest reliability of ACC measures (Tremblay et al., 2004; Kim, 2015; Meehan et al., 2024), and the present findings extend this evidence by showing that such stability is preserved even in a continuous speech paradigm. This suggests that ACC responses reflect stable cortical processing characteristics rather than transient neural variability.

Most previous ACC studies have employed discrete stimuli with long inter-stimulus intervals to isolate neural responses to acoustic changes (Friesen and Tremblay, 2006; Brown et al., 2015). While these paradigms provide strong experimental control, they are time-consuming and do not reflect the continuous nature of everyday speech perception.

The present study demonstrates that ACC responses can be elicited reliably in a continuous, pause-free speech sequence, thereby extending prior work into a more ecologically valid framework. This is particularly relevant given that natural speech perception requires continuous tracking of rapid acoustic changes. From a neurophysiological perspective, this aligns with models emphasizing dynamic cortical updating and predictive processing during ongoing auditory input (Näätänen et al., 2007; Bendixen et al., 2012).

Importantly, this work addresses a key limitation highlighted in recent literature, namely that ACC paradigms are often too time-intensive for routine clinical implementation (Meehan et al., 2024). By demonstrating robust ACC responses within a short recording time using a simplified electrode configuration, the present study provides evidence that clinically efficient ACC protocols are feasible.

Previous studies have reported variable relationships between ACC measures and behavioral speech perception outcomes. While some studies demonstrate meaningful correlations between ACC amplitude and perceptual discrimination (Won et al., 2011; Brown et al., 2017), others have reported limited predictive value of ACC responses for speech perception performance in cochlear implant users (Brown et al., 2015). These discrepancies suggest that ACC responses primarily reflect neural discrimination capacity at the cortical level, whereas speech perception involves additional cognitive and linguistic processes.

Importantly, recent work examining auditory training in cochlear implant users demonstrated that behavioral improvements in spectral and pitch discrimination may occur without corresponding changes in ACC responses, and without significant correlations between electrophysiological and behavioral outcomes (Jeon et al., 2025b). These findings further support the interpretation that ACC reflects cortical encoding of acoustic change but may not be sufficiently sensitive to detect short-term or moderate perceptual gains. The present findings are consistent with this perspective, as they demonstrate robust cortical discrimination under controlled conditions while highlighting the need for careful interpretation of ACC as a functional outcome measure.

Although quantitative differences in ACC responses across phoneme transitions were observed using RMS measures, formal inferential statistical comparisons were beyond the scope of this feasibility study. Future studies with larger sample sizes will be important to further evaluate transition-dependent effects.

The clinical implications of these findings are substantial. Unlike peripheral objective measures such as electrically evoked compound action potentials (ECAPs) or electrically evoked stapedius reflex thresholds (ESRTs), which primarily reflect early auditory pathway function, ACC responses provide a direct measure of cortical discrimination processes that are more closely related to functional listening abilities (Jeon et al., 2010; Kim, 2015; Sanju et al., 2023; Ching et al., 2023; Meehan et al., 2024).

The use of Ling-six stimuli further enhances clinical applicability. These phonemes are widely used in clinical practice to assess access to the speech frequency spectrum and to monitor functional listening. By integrating Ling-six stimuli into an electrophysiological paradigm, this approach bridges the gap between objective neural measures and clinically meaningful speech sounds.

Additionally, prior work has demonstrated that ACC responses are sensitive to changes in listening conditions and auditory input. For example, ACC amplitudes vary as a function of listening mode in hybrid cochlear implant users, reflecting functional differences in auditory processing and benefit (Jeon et al., 2023). This supports the potential use of ACC as an objective tool for evaluating device benefit and guiding individualized clinical management.

Furthermore, ACC responses are influenced by auditory experience and cortical plasticity. Brown et al. (2017) demonstrated enhanced ACC responses in individuals with long-term musical training, indicating experience-dependent cortical changes. Developmental work has also shown that cortical auditory evoked potentials, including ACC responses, reflect maturation of central auditory pathways and are influenced by noise and listening conditions (Jeon et al., 2025a). Together, these findings support the potential use of ACC as an objective biomarker for monitoring auditory development and cortical plasticity.

However, it is important to recognize the limitations of ACC as a clinical tool. As demonstrated in recent auditory training studies, improvements in perceptual performance may not be accompanied by measurable changes in ACC responses (Jeon et al., 2025b). This suggests that ACC should not be interpreted as a direct proxy for functional speech perception or rehabilitation outcomes, but rather as a complementary measure of cortical discrimination capacity.

Finally, the passive nature of the recording paradigm makes this approach particularly valuable for difficult-to-test populations, including infants, young children, and individuals with additional disabilities. ACC responses have been successfully recorded in such populations, supporting their role as an objective measure of speech discrimination capacity (He et al., 2015; Meehan et al., 2024).

## 5 Limitations

Several limitations should be considered. First, the study included a relatively small sample of normal-hearing adults, limiting generalizability to pediatric and hearing-impaired populations. Second, behavioral measures of speech discrimination were not directly correlated with ACC responses, limiting interpretation of functional significance. Third, the use of a fixed phoneme sequence may introduce predictability effects that could influence cortical responses.

Fourth, the study did not include a control condition using a single stimulus with controlled acoustic changes (e.g., amplitude-modulated vowel stimuli), which has been used in prior ACC studies to directly quantify the relationship between acoustic change magnitude and neural response.

Additionally, while continuous paradigms improve ecological validity, they may introduce greater variability in response morphology, which could complicate waveform interpretation and highlight the need for more objective and automated analysis methods.

## 6 Future directions

Future research should extend this paradigm to pediatric and cochlear implant populations, where objective measures of speech discrimination are critically needed. Establishing relationships between ACC responses and behavioral speech perception outcomes will be essential to validate clinical utility.

Further work is also needed to optimize stimulus design, including phoneme selection and sequence variability, and to develop automated analysis approaches to improve reliability and clinical feasibility. Given evidence that electrophysiological and behavioral measures may diverge, future studies should also examine factors influencing this relationship, including training duration, stimulus complexity, and individual variability.

Longitudinal studies examining changes in ACC responses over time will be particularly important for understanding auditory development, cortical plasticity, and intervention outcomes.

## 7 Conclusion

In summary, this study demonstrates that continuous Ling-six speech stimuli can reliably evoke ACC responses reflecting cortical speech sound discrimination. By combining ecological validity, efficiency, and clinical relevance, this paradigm represents an important step toward the development of objective tools for assessing auditory discrimination and monitoring auditory learning in clinical populations.

## Data Availability

The raw data supporting the conclusions of this article will be made available by the authors upon reasonable request.
